# Supplementary material for: Are carbon emissions trading and green financial instruments synergistic? -Comprehensive quantitative research based on content analysis
Source: PLoS One. 2024 Mar 7;19(3):e0298601. doi: 10.1371/journal.pone.0298601 (PMC10919601; doi:10.1371/journal.pone.0298601)
Supplement: S1 Data — (ZIP) [file pone.0298601.s001.zip › Data/地级市Z_F工作报告.zip!/║╙─╧/╚2├┼╧┐╩╨/└┤╫╘╣1⁄2╓┌║┼í╛┬φ┐╦╩2╛▌═°í┐_2017.docx]

各位代表：

现在，我代表市人民政府，向大会报告工作，请予审议，并请各位政协委员和其他列席人士提出意见。

一、本届政府及2016年工作回顾

2012年本届政府任期以来，在省委、省政府和市委的坚强领导下，在市人大、市政协的监督支持下，面对一波三折的宏观经济形势，面对稳增长、调结构的双重压力，我们主动适应经济发展新常态，培育新动力，谋求新作为，拓展新空间，迈出了全面建成小康社会的坚实步伐。

五年来，我们扭住发展主线，稳增长备后劲，综合实力迈上新台阶。全市生产总值突破千亿大关，财政一般公共预算收入突破百亿，分别是2011年的1.3倍、1.7倍，年均增长8.2%、11.9%；全社会固定资产投资是2011年的2.3倍，年均增长17.8%；社会消费品零售总额是2011年的1.8倍，年均增长13%，综合经济实力实现了新跨越。

五年来，我们坚持产业为基，调结构增效益，转型升级实现新突破。三次产业结构由2011年的7.9∶68.1∶24优化至9.1∶57.4∶33.5，经济结构更趋合理。第一产业增加值年均增长4.7%，走在全省前列；规上工业增加值年均增长9%；第三产业增加值占GDP比重比2011年提高9.5个百分点，成为稳增长、扩就业的生力军。

五年来，我们狠抓基础建设，强载体建平台，发展支撑得到新增强。三淅高速、郑卢高速等一批高速公路项目建成，蒙华铁路等一批铁路项目加快推进，区域性交通枢纽初步形成。三门峡海关、出入境检验检疫局、国家铝及铝制品质检中心、国家果品及果蔬汁检测重点实验室、省黄金贵金属产品质检中心等通关检测机构建成。全市7个产业集聚区累计完成投资3031亿元，年均增长29%，成为推动全市经济增长的主阵地。服务业“两区”建成区面积5.82平方公里，入驻企业1014个，从业人员5.7万人，成为服务业扩量提质的新引擎。

五年来，我们聚焦城乡统筹，拉框架提功能，城乡建设掀开新篇章。《三门峡市城市总体规划（2013—2030）》编制完成，陕州区正式挂牌，中心城区建成区面积71平方公里，人口达到65万，城市功能更加完善，承载能力明显增强；义渑一体化步伐加快，灵宝市、卢氏县城区框架拉大，带动力持续增强；一批重点城镇快速发展，打造美丽乡村示范村204个，初步形成了中心城区、县城、中心镇、美丽乡村协调发展的新型城镇化格局。全市城镇化率53.1%，高于全省4.6个百分点。

五年来，我们加快动能转换，抓改革促开放，经济发展注入新动力。“放管服”改革、经济体制改革、农村改革等重点领域改革稳步推进，国有企业、政府机构、医药卫生等改革取得阶段性成效。抢抓国家“一带一路”战略和晋陕豫黄河金三角区域合作上升为国家战略机遇，持续扩大开放合作，累计引进省外资金1454亿元，年均增长16.1%；实际利用外资46.5亿美元，年均增长11%。深入实施创新驱动战略，全市高新技术企业达到45家，科技型中小企业达到111家，建成省级院士工作站16家，省市级研究中心71家，科技进步对经济增长的贡献率达到53.9%。

五年来，我们注重以人为本，办实事惠民生，群众获得感得到新提升。城乡居民人均可支配收入分别达到25254元和11982元，年均增长8.6%和11.3%，是2011年的1.5倍和1.7倍，农村居民人均可支配收入增速连年位居全省前列。脱贫攻坚深入推进，累计实现16.9万贫困人口稳定脱贫，完成易地搬迁40665人，实施整村推进140个，贫困地区基础设施和公共服务水平大幅提升。城镇新增就业28万人，城镇登记失业率连年控制在3%以下。社会保障制度不断完善，企业退休人员养老金稳步增长，城乡低保标准分别提高53.8%、90.6%。社会事业全面进步，崤函古道成功“申遗”，本科教育实现“零”突破，市中心医院、市中医院晋升“三甲”。生态文明建设成效显著，社会大局和谐稳定。

各位代表！五年来的艰辛奋斗，我们深深感到，做好政府工作：必须坚持党的领导，始终在思想上政治上行动上同以习近平同志为核心的党中央保持高度一致，同省委、市委保持高度一致，坚定正确的政治方向。必须坚持以人为本，牢固树立以人民为中心的发展思想，始终做到发展为了人民，发展依靠人民，发展成果由人民共享。必须坚持发展为要，把发展作为解决一切问题的关键，加快发展，提升发展，扩大总量，壮大实力。必须坚持依法行政，强化法治理念和法治思维，坚持法无授权不可为，法定职责必须为，重大决策事项及时向人大及其常委会报告，依法规范权力运行，努力建设法治政府。必须坚持改革创新，把破除体制机制障碍作为加快发展的根本动力，在改革创新中集聚动能、释放活力。这些实践经验，我们将长期坚持并发扬光大。

2016年，是本届政府最后冲刺的收官之年，也是承上启下、开创局面的关键之年。我们认真落实省委、省政府和市委各项决策部署，全力以赴稳增长、调结构、促改革、惠民生，全市经济全面企稳回升，底线构筑更加坚实，重大项目全面发力，干成了一批打基础利长远的大事要事，取得了系列具有标志意义的突破，实现了“十三五”良好开局。全市生产总值完成1348.6亿元，同比增长7.7%；财政一般公共预算收入完成100.1亿元，增长7.8%；规上工业增加值完成616亿元，增长7.3%；全社会固定资产投资完成1783亿元，增长15.1%；社会消费品零售总额完成441.1亿元，增长11.2%。

2017年一季度，全市经济持续回升，多项指标跃居全省前列，实现了首季“开门红”。生产总值增长8.5%，居全省第5位，其中第一产业增加值增长4.2%，居全省第1位；规上工业增加值增长9.1%，居全省第2位；财政一般公共预算收入增长12.8%，居全省第8位；进出口总值增长77.8%，居全省第2位。

（一）重大项目和重点工作实现新突破。坚持重点带动，一批事关全局的大事要事取得重要突破。“三大战役”扎实顺利推进。脱贫攻坚首战告捷，全市精准脱贫34075人，超额完成省定目标，易地搬迁19484人，35个贫困村退出，荣获全省脱贫工作成效考核第1名；大气污染防治有力有效，PM2.5指标超额完成省定目标，空气质量明显改善，优良天数大幅增加；国企改革不断深化，“三供一业”剥离移交等工作稳步推进。重大项目建设实现重要突破。全市七批共69个项目争取国家专项基金67.3亿元，居全省第3位；20个省重点项目和242个市重点项目分别完成投资147.1亿元、652.7亿元，占年度目标的122.2%和118.9%，为经济发展积蓄了巨大后劲。

速达纯电动汽车项目获得国家发改委核准，全国首批、全省首家新能源汽车牌照落户三门峡。投资116亿元的国道310南移项目全线开工，是我市建市以来投资最大的项目。蒙华铁路、垣渑高速等项目进展顺利，陇海铁路取直改线、运三高铁、三洋铁路等项目取得重要突破，使我市朝着高铁、高速“双高中心”和客运、货运中心迈出了实质步伐。央地合作迈出重大步伐。与中国兵器集团等12家央企签订19个项目合作协议，总投资333.2亿元，为经济发展拓展了新空间。高等教育实现历史突破。三门峡社会管理职业学院、三门峡技师学院一次性获得省政府批准，三年内将形成聚集4万人的高等职教园区，成为助推新型城镇化建设，加快产业转型升级新动能。重大改革创新取得重要进展。深化投融资体制改革，整合成立市投资集团公司，初步建成了融资能力近期超千亿、远期超5000亿元的融资平台。新型智慧城市建设全面启动。

（二）供给侧结构性改革不断深化。持续推进种养业、制造业和服务业供给侧结构性改革，三次产业协调发展。特色农业持续壮大。第一产业增加值增长4.7%，增速居全省第1位。果品、食用菌、烟叶等特色产业持续提升，省市农业产业化集群达到39个，规模以上农业龙头企业达到218家，运行良好的农民专业合作社达到1400家，“三品一标”农产品数量达138个，“二仙坡”荣获“中国驰名商标”。涉农电商突破2100家，农产品电商交易额突破10亿元，全省农业电子商务发展现场会在我市召开。工业经济提质增效。实施亿元以上工业转型升级项目53个，完成投资272.8亿元，骏通车辆多功能起重举升专用车、义腾新能源动力锂电池隔膜、宝鑫电子专用铜箔等项目进展顺利，德道电子汽车电子产品等项目建成投产，为加快先进制造业发展奠定了坚实基础。服务业扩量提质。第三产业增加值增长9.1%，占GDP的比重达到33.5%，比上年提高1.2个百分点。三门峡电商产业园、跨境电商产业园投入运营，全市电商主体达到4522家，交易额突破42亿元。成功举办第22届旅游节暨第4届特博会，成功举办中国（三门峡）白天鹅·野生动物国际摄影大展，天鹅湖湿地公园、陕州地坑院受到中央主流媒体广泛关注，“天鹅之城”品牌更亮、人气更旺。全市接待海内外游客3028万人次，旅游总收入258.2亿元，分别增长12.7%和14.5%。

（三）“去降补”工作成效显著。坚定不移去产能。化解煤炭行业过剩产能36万吨，涉及矿井已关停到位。综合施策去库存，全市商品房库存面积229.6万平方米，比年初减少77.7万平方米。服务企业降成本，实施降成本专项行动，9家企业进入省电力交易平台，为企业降低用电成本1540万元；建立市县两级信用担保服务体系，6家政策性担保机构注册资本达到3.6亿元。因地制宜补短板，持续强化基础设施、创新能力、人力资源等方面建设，大石涧水库等一批重大基础设施项目进展顺利，市级以上科技创新平台达到136个，引进培养中高级人才4113人。

（四）区域中心城市建设加快。以新型智慧城市建设为总抓手，拉大框架，做优服务，做强功能，做旺人气，区域中心城市建设取得新进展。新型智慧城市建设全面启动。顶层设计初步完成，运营公司注册成立，“一平台、两中心、三应用”项目有序推进，朝着“管理一块屏，服务一张网，决策一组数”新型智慧城市建设迈出了新步伐。城市空间布局更加优化。陕州区撤县设区，三门峡进入“一城两区”时代；商务中心区完成投资75亿元，“五纵五横”道路框架基本形成，城市新中心形象初显；城乡一体化示范区和三门峡产业集聚区套合完成，管理体制更加规范。湖滨区、陕州区、商务中心区、城乡一体化示范区联动发展格局初步形成。城市功能品位持续提升。大岭北路、崤山路等11条道路打通和提升工程完工，新增城市供热面积50万平方米，黄河公园与陕州公园连接带、青龙涧河景观带改造提升项目建成。洁城行动稳步推进，城市环境和秩序明显改善，通过省级卫生城市复审。旧城区和城中村改造进展顺利。

（五）发展活力不断增强。坚持全面深化改革开放，激发转型发展动力活力。重点领域改革深入推进。扎实推进“放管服”改革，行政审批事项精简幅度居全省前列；营改增试点进展顺利，预算管理制度改革不断深化；土地确权登记颁证等农村改革持续推进；商事制度改革成绩突出，受到国务院表彰，“智慧工商”大数据经验在全省推广。经济发达试点镇、医疗卫生、机关事业单位养老、城乡居民医保整合、公务用车等领域改革取得新进展。开放合作不断扩大。突出培育优势产业，围绕打造黄金产业链、铝及铝精深加工、旅游业、现代物流、电子商务、地区性交通枢纽深化招商，签约招商项目142个，总投资607.1亿元。实际利用外资10.6亿美元，增长5.4%；实际到位省外资金352.2亿元，增长7.6%。进出口逆势上扬，增长155.8%，增速居全省第1位。黄河金三角区域合作迈出新步伐，省级协调机制和市级推进机制初步建立，基础设施、产业发展和公共服务等领域合作不断深化，三门峡市与运城市平陆县城际公交专线顺利开通。政银企合作成效显著。先后与省农发行、国开行、建行签订总金额1500亿元的战略合作协议，协调金融机构为121个项目贷款321亿元，金融对实体经济的支持力度进一步增强。

（六）群众生活持续改善。坚持把保障改善民生作为政府工作的出发点和落脚点。民生投入不断加大。“十项重点民生工程”持续推进，全市财政民生支出137.5亿元，占一般预算支出的73.6%。就业创业扎实推进。深入实施大众创业万众创新，认真落实稳岗扶持政策，全市城镇新增就业6万人，失业人员再就业2.3万人，城镇登记失业率2.82%。社会保障稳步提升。城乡低保年补助标准分别上调600元和450元，城镇居民医保年人均补助标准提高到420元，城镇居民大病保险制度、农村居民重大疾病保障机制进一步完善。企业和机关事业单位退休人员基本养老金调整到位。社会事业不断进步。“全面改薄”、城镇义务教育资源扩充计划扎实推进，第二期学前教育三年行动计划全面完成，引进江苏教育集团清华园学校项目开工。城市公立医院综合改革进展顺利，妇幼保健院和儿童医院开工建设，全面二孩政策稳妥实施。《文化三门峡》丛书初稿编撰完成，“2016中国·三门峡横渡母亲河”活动被授予中国“体育旅游精品赛事”称号。精神文明成果丰硕。黄河公园、湖滨区涧南社区、灵宝河西村入选河南省首批社会主义核心价值观建设示范点，未成年人思想道德建设、志愿者服务和农村精神文明创建活动扎实开展。社会大局保持平稳。预防和化解社会矛盾，全面排查处置非法集资风险，信访形势总体平稳。持续加强安全生产和食品安全监管，平安三门峡、法治三门峡建设扎实推进，群众安全感持续提升。

扎实开展“两学一做”学习教育，驰而不息纠正“四风”，政府自身建设不断加强。主动接受市人大及其常委会监督，支持市政协履行职能，认真办理人大代表建议184件、政协委员提案230件。双拥共建再创佳绩，荣获全国双拥模范城“五连冠”，军民融合有效推进，军政军民团结更加巩固。统计、审计、外侨、民族宗教、对台、史志、人防、气象、防震减灾、无线电、黄河河务、妇女儿童、残疾人、老龄、慈善等工作取得新成绩。

各位代表！市政府各项成绩的取得，是省委省政府和市委正确领导的结果，是市人大、市政协大力支持的结果，是全市人民团结拼搏的结果。在此，我代表市人民政府，向全市各行各业的建设者，向各位人大代表、政协委员、各民主党派，向驻峡解放军、武警官兵、公安民警，向广大客商以及关心支持三门峡发展的各界人士，致以崇高的敬意和衷心的感谢！

在看到成绩的同时，我们也清醒地认识到，目前经济社会发展仍存在不少矛盾和问题：经济发展水平不高的基本市情还没有根本改变，总量不大，综合实力不够强；传统产业占比偏高，新兴产业培育不快，结构不优，特色优势不明显，区域竞争力不够强；农村贫困人口量多面广，脱贫攻坚任务依然艰巨，生态建设、环境保护、安全生产等方面仍存在薄弱环节，转型发展攻坚任务较重；城乡公共服务和基础设施保障等方面欠账较多，与群众期望仍有差距，民生改善任务较重；政府依法行政水平和政务服务效能有待进一步提高，少数公务员懒政怠政等问题仍然存在，优化发展环境任务较重等。对此，我们将在新一届政府任期内，采取有效措施，切实加以解决，决不辜负全市人民的重托！

二、今后五年政府工作的总体要求和奋斗目标

未来五年，是我市全面建成小康社会的决胜期，是实现经济社会转型发展的攻坚期，也是加快建设省际中心城市的机遇期。从外部环境看，国内宏观经济总体平稳的大环境没有改变，国家“一带一路”战略、河南省“三区一群”和晋陕豫黄河金三角区域合作战略的深入实施，将为我们加快转型发展提供强有力的政策叠加机遇。从自身条件看，多年发展打下的基础扎实，项目支撑有力，未来转型发展的方向明确、目标清晰，特别是全市上下人心思稳、人心思干、人心思进的局面正在形成，敢于担当、干事创业的合力加速凝聚，为我们加快发展提供了坚实的产业基础、群众基础和思想基础。机遇承载使命，责任考验担当。只要我们坚定信心、团结拼搏，经过不懈奋斗，就一定能够开创转型发展新局面！

今后五年工作的总体要求是：高举中国特色社会主义伟大旗帜，以邓小平理论、“三个代表”重要思想和科学发展观为指导，深入贯彻落实习近平总书记系列重要讲话精神，按照“五位一体”总体布局和“四个全面”战略布局，落实新发展理念，紧紧围绕建设省际中心城市这一目标，围绕提升区域核心竞争力这一根本，加快转型创新发展，加快推进“四个大市”建设，以“三次创业”凝聚人心士气，以“效能革命”激发动力活力，推动经济持续健康发展和社会和谐稳定，决战决胜打赢脱贫攻坚战，奋力夺取全面建成小康社会伟大胜利。

今后五年的发展目标是：

全面建成小康社会目标圆满完成。在提高发展质量效益的基础上，主要经济指标增速高于全省和黄河金三角地区平均水平。地区生产总值年均增长8%以上；全社会固定资产投资年均增长、财政一般公共预算收入年均增长均超过全省平均水平，人均指标继续走在全省前列。农村贫困人口全部脱贫，贫困村全部退出，卢氏县摘帽脱贫。物质文明、精神文明、政治文明和生态文明共同进步。

转型创新发展实现重大突破。经济结构战略性调整取得显著成效，特色农业持续壮大，新型工业化加速推进，现代服务业扩量提质，网络经济快速发展，努力建设新型工业化城市、高成长性服务业先进市、特色农业强市、网络经济大市，初步实现新型工业化、新型城镇化建设的历史任务。

省际中心城市建设取得实质性进展。“双百战略” 深入实施，城市框架显著拉大，传统优势产业和战略新兴产业不断壮大，初步建成“三地五中心”，即中西部地区重要的先进制造业基地、全国重要的大宗商品物流基地、全国知名的旅游目的地和全国有影响的行业数据中心、大健康中心，中西部地区重要的黄金珠宝加工交易中心，黄河金三角地区客运中心和高等职业教育中心。城市的集聚、辐射、带动功能不断增强，在黄河金三角地区的引领作用进一步凸显。

区域核心竞争力全面增强。传统产业改造升级加快，新产业、新动能持续凝聚，产业实现链条由“价值链低端”向“价值链中高端”转变，产业竞争力持续增强；文化资源深度开发，文化产业创新发展，基本建成华夏历史文明传承创新区重要板块，文化竞争力持续增强；生态文明建设迈上新台阶，天更蓝、水更清、地更绿、城市更宜居，生态竞争力持续增强；努力营造公平竞争的市场环境、安全放心的消费环境、守法诚信的发展环境和廉洁高效的政务环境，新型智慧城市建成运营，服务竞争力持续增强，全面增强区域核心竞争力。

人民生活水平显著提升。基本公共服务均等化水平稳步提高，公共服务体系更加健全，社会保障体系进一步完善，人民健康水平持续提升，教育事业全面进步。收入分配制度不断完善，城乡居民收入普遍较快增长，城乡收入差距逐步缩小。人民群众精神文化生活更加丰富，创造活力进一步激发。改革发展成果更多更公平地惠及全市人民，社会治理能力和水平进一步提高，人民群众的安全感、获得感、幸福感明显增强。

三、2017年政府工作的主要目标和任务

今年是实施“十三五”规划的关键之年，是推进供给侧结构性改革的深化之年，也是全面落实省十次党代会、市七次党代会各项部署的开局之年。做好今年政府工作，对于巩固发展良好态势，全面推进各项事业，维护社会大局稳定至关重要。

根据市委经济工作会议部署，今年经济社会发展的主要预期目标是：生产总值增长8%以上，财政一般公共预算收入增长8%，全社会固定资产投资增长12%左右，社会消费品零售总额增长11%左右，进出口总值保持稳定，城乡居民人均可支配收入分别增长8%和9%左右；节能减排、环境保护等约束性指标完成省定任务，发展质量和效益明显提高，供给侧结构性改革取得实质性进展。

重点抓好八个方面工作：

（一）加大招商引资力度，强化项目建设，促进经济稳定增长。紧紧围绕发展壮大优势产业，以大招商引进大项目，以大项目带动大发展。

强化招商引资扩增量。围绕发展和壮大优势产业，重点打造以铝精深加工和新能源汽车为主的装备制造产业园、黄金珠宝首饰加工产业园、铜精深加工产业园、大数据产业园、物流产业园，紧紧盯住重点央企、大型民企、新兴市场主体，招引一批科技含量高、市场前景好、产业带动强的项目。积极适应招商形势变化，强化协会招商、网络招商、以商招商，提高招商实效。压实工作责任，加强落地项目服务，重点项目实行“五个一”推进机制，切实提高签约项目“三率”。

抓好项目建设强基础。坚持以项目建设为抓手，着力扩大有效投资，重点抓好240个省、市重点项目，年度完成投资580亿元以上。持续推进速达纯电动汽车项目建设，确保按时顺利通过工信部生产准入，加快建设500亿级新能源汽车产业集群。加快推进同人铝业铝合金铸造及深加工、戴卡轮毂铝合金汽车轮毂项目，力争早日投产；抓好凯斯曼（三门峡）汽车零部件项目前期工作，争取早日开工。深入落实国家和省一系列鼓励民间投资的支持政策，顺应国家有序放开配电网、电信、市政、社会服务等领域市场准入，谋划研究一批符合民间资本和各类产业基金“胃口”的好项目，吸引更多民间资本进入。

稳定经济运行保基本。加强经济运行调控，建立土地、资金等重点要素与外部市场联动调节机制。突出抓好企业服务，完善市领导分包、“首席服务员”等制度，持续搞好产销、银企、用工、产学研“四项对接”，加大对黄金、电解铝、煤化工、装备制造等困难行业的政策和信贷支持力度，实现主导产业经济运行平稳。切实防范风险隐患，妥善应对不良资产、企业互保等风险，严厉打击和处置非法集资，有效防控金融市场风险；引导企业强化风险意识，加强财务管控，优化债务结构，降低企业杠杆，稳健有序发展，有效防控企业经营风险；推动政府融资机制规范化、透明化和法制化，有效防控债务风险。

（二）加快结构转型升级，提高供给体系质量和效率。加大供给侧结构性改革力度，努力走出一条结构优、效益佳、活力足、后劲强的发展路子。

持续加快“去降补”任务落地。把“三去一降一补”作为供给侧结构性改革的硬任务和转型发展的着力点持续推进。去产能，合理安排产能退出时序和进度，做好14个煤矿的退出工作，妥善处置“僵尸企业”，用市场、法治的办法做好去产能工作。去库存，因城施策、分类指导调控房地产市场，通过加大棚户区改造货币化安置、鼓励农民进城购房、扩大公积金受益覆盖面等措施，消化房地产库存。去杠杆，把降低企业特别是国有企业杠杆率作为重中之重，盘活企业存量资产，提高直接融资比例，探索不良资产收益权转让试点，通过债转股、资产重组、上市融资等方式实施债务重组，优化负债结构。降成本，深入落实国务院降成本工作方案和省定50条措施，积极推动减税、降费、降要素成本，同时推动企业“眼睛向内”降本增效。补短板，重点抓好投资100亿元的国家储备林基地建设、投资122亿元的城中村和棚户区改造、投资65亿元的城市生态水系工程等薄弱领域的重大项目，补齐经济社会发展短板。

持续推进供给侧结构性改革。加快产业结构调整，不断提升产业核心竞争力。强化工业经济“双轮驱动”，围绕提升中高端加工、迈向中高端水平这一目标，强化传统优势产业升级、接续替代产业培育“双轮驱动”，一手抓黄金、铝、煤化工等传统产业提升发展，着力激活传统动能，一手抓新能源汽车、装备制造、铜精深加工、特种机器人、食品、生物医药等战略性新兴产业创新发展，着力打造经济增长新引擎。加快现代服务业提质转型，重点抓好现代物流、黄金珠宝加工交易、金融保险、信息服务、文化旅游和健康养老等六大主导产业发展，深入推进农业电子商务发展三年行动计划，促进电子商务持续发展。增加特色农产品优质供给，加强地理标志保护产品开发、保护和利用，生产更多具有生态属性的中高端农产品。以苹果为突破，打造农业生产物联网，完善农产品标准质量体系。加快推进农村一、二、三产业融合发展，以寺河山万亩果园、二仙坡苹果基地为龙头，促进观光农业、加工农业、体验农业、乡村旅游有机融合发展。

持续加强科学发展载体建设。坚持产业集聚区发展定位，突出壮大主导产业集群优势，着力破解产业配套、融资支持、人力资源、土地保障、管理体制等瓶颈制约，持续推进产业集聚区“三上一提高”。全市产业集聚区实现规上工业增加值增长10%左右，主营业务收入增长10%左右，固定资产投资增长13.5%以上。持续推进商务中心区扩容提质，年内完成投资100亿元以上，促进与陕州区、湖滨区及相关区域有机融合，实现联动发展；加快招商项目建设、盘活问题项目，推动各类优质教育、医疗资源汇集，培育打造生产性服务业集聚中心。加快推进特色商业区设施完善和产业提升，重点抓好高阳山国际温泉度假区、万洋国际商贸博览城等一批龙头项目，打造优势服务业产业集群。加快城乡一体化示范区建设，完成总体规划评审，先行启动一批基础设施和产业项目。

（三）着力建设“三地五中心”，培育转型发展新优势。依托区位交通、自然资源、文化旅游等方面的比较优势，着力打造“三地五中心”，为建设省际中心城市提供有力支撑。在先进制造业基地建设上，依托现有产业基础，重点打造汽车、黄金、铝工业、煤及煤化工四大全产业链，着力打造装备制造产业园、珠宝首饰加工产业园、铜精深加工产业园、节能环保产业园、大数据产业园、中加科技产业园、中国—以色列光电产业园，加快培育先进装备制造业，改造提升水工机械、化工机械、矿山机械等传统制造业，积极发展量仪、白刚玉等制造业配套产业，努力打造中西部地区重要的先进制造业基地。在物流基地建设上，依托陇海、蒙华、三洋三大国家干线铁路交会优势和金精粉、铜精矿大进大出实际，深化与杭州锦江集团等大企业合作，加快推进大宗商品物流产业园项目，建设“物流云”和物流行业数据中心，积极推动冷链、保税、电子商务等行业物流园区建设，努力建设全国重要的大宗商品物流基地。在旅游目的地建设上，依托旅游资源优势，积极与深圳华侨城、旅游百事通等国内大型旅游集团合作，着力引爆天鹅湖、地坑院、三门峡大坝、豫西大峡谷、函谷关等核心景点，打造精品线路，发展智慧旅游，形成“点、线、面”有机结合的全域旅游发展新格局，努力建设全国知名的旅游目的地城市。在行业数据中心建设上，依托气候凉爽、地质稳定、电力充足等适宜数据机房建设的优势，与国内外知名互联网企业进行深度合作，加快谋划建设区域性大数据中心，努力打造全国有影响的特色农产品、黄金行业数据中心。积极培育发展智能装备、信息感知、移动终端、新型显示、智能应用等大数据端产品产业，以大数据引领业态创新和产业升级。在黄金珠宝加工交易中心建设上，依托黄金资源和精炼金产能优势，整合小秦岭等区域黄金资源，加快金源矿业、灵宝黄金股份等黄金企业改造升级，加快建设三门峡黄金珠宝交易中心、加工中心，打造特色黄金小镇，推动黄金珠宝、黄金工业品加工、黄金交易等后续产业发展，打造黄金全产业链，建设中西部地区重要的黄金珠宝加工交易中心。在大健康中心建设上，依托森林覆盖率高、负氧离子多、温泉品质好等自然资源优势和道家养生文化等文化资源优势，着力推动陕州高阳山、翡翠丽池温泉扩大规模、提升层次，增强陕州地坑院品牌影响力，逐步把品质独特的自然、文化优势发挥出来，努力打造全省乃至全国有影响的休闲度假中心。推动市中心医院、黄河医院、中医院做优做强重点专科，积极推动与郑大一附院、省中医药大学等合作，建设集医疗、康复、保健、度假、养老为一体的新型医养中心。加快妇幼保健院和儿童医院建设，推动与互联网医疗公司合作，建设高水平的智慧医院和互联网医院，努力打造黄河金三角地区医疗服务中心。在客运中心建设上，积极推进运十高铁、运三高速、垣渑高速、栾卢高速等项目建设，加快形成高铁、高速“双高”交会格局；抢抓国家低空空域开放机遇，谋划推进三门峡通用机场、通用航空产业园等项目，努力打造黄河金三角地区客运中心。在高等职教中心建设上，依托三门峡职业技术学院，积极创建国家优质高职院校，加快独立设置本科院校建设，全面启动职业技术学院新校区建设；进一步扩大河南科技大学应用工程学院招生规模，力争今年突破400人；积极抓好三门峡社会管理职业学院、三门峡技师学院招生工作；深化与中国黄金集团合作，推动黄金工业学校升格为三门峡黄金学院，推动建设一所本科、四所大专的高等职业教育格局，尽快形成聚集4万人左右的职教园区，努力打造黄河金三角地区高等职业教育中心。

（四）科学推进新型城镇化，初步建成新型智慧城市。顺应我省推进国家大数据综合试验区建设趋势，抢抓从IT（信息技术）时代转向DT（大数据）时代契机，全面贯彻落实国家、省新型城镇化相关部署，统筹规划，一体建设，在线管理，不断加快推进新型智慧城市建设，努力实现“一年打基础、两年出形象、三年成先进”的目标。年内建成运行“一平台、两中心、三应用”，即云计算平台，城市运营指挥中心、大数据中心，移动协同办公系统、智慧城管、互联网+政务，形成新型智慧城市的基本框架。

统筹规划。以政用、民用、商用为主线，以“优政、惠民、兴业”为目标谋划好新型智慧城市建设，不断推动互联网与政府治理深度融合，与便民服务深度融合，与实体经济深度融合，与城市规划建设深度融合，全面提升城市治理现代化和人性化水平。政用方面，建设完成移动协同办公系统、智慧城管和人口库、法人库、宏观经济库等重要数据库，做到用数据说话、用数据决策、用数据管理、用数据创新、用数据发展，推进政府决策科学化、社会治理精准化、公共服务高效化。民用方面，建设完成互联网+政务服务，推动重点公共区域免费无线WiFi全覆盖，推进医疗、教育、社保等智慧服务建设，让数据多跑路，让群众少跑腿，提升人民群众的幸福感。商用方面，围绕企业对数据的需求，大力推进数据资源汇聚共享开放，构建大数据产业发展生态，激发市场主体活力，鼓励和支持社会力量参与大数据基础设施建设和大数据创新应用，促进大数据专业化、市场化发展，推动政府、企业、社会共采“数据金矿”，实现多方共赢发展。

一体建设。坚持地下地上一体推进，线上线下一体推进，智慧城市建设和大数据产业培育一体推进，实践探索和制度创新一体推进，不断加大投入力度，积极推进相关职能整合、项目整合和数据整合，统筹实施新型智慧城市建设“四个一”工程，即全市联通“一张网”，数字空间“一张图”，数据产业“一个园”，建设运营“一套制度体系”，形成软硬结合、远近结合、四位一体、系统完备的新型智慧城市建设体系。“一张网”，就是推动互联网、专网、物联网建设，加快建设全市云平台和大数据中心，推广县（市）城区光纤接入，积极开展4G+技术普及应用，推动区域互联、万物互联、人人互联，拓展网上空间，建设“网上三门峡”。“一张图”，就是地下加快推进海绵城市、综合管廊、人防工程建设，地上持续完善提升城市基础设施水平，扎实推进五原中路等12条城市道路、商务中心区供热管网等基础设施项目，尽快开工城市生态水系、青龙谷公园等重点工程，同步部署和采集相关数据信息，实时收集相关数据，着力打造城市地上地下空间数据库，形成“图上三门峡”。“一个园”，就是建设大数据产业园，坚持以特色农业大数据、物流大数据、黄金大数据等行业数据中心建设和大数据应用为基本方向，注重政府与市场协同发力，与华为、中兴、浪潮、启明星辰等国内知名互联网企业深度合作，积极开展大数据深度挖掘和创新应用，引进和培育一批具有行业领先地位的大数据企业，形成一批具有自主知识产权的大数据产品，建成一批有较强辐射带动力的大数据关联业态，努力打造全省一流的大数据产业中心、大数据应用先导区、大数据企业集聚区和制度创新先行区，为全市转型发展提供新动能。“一套制度体系”，就是坚持自主可控可持续的新型智慧城市建设道路，积极开展体制机制创新。在领导体制上，加快组建大数据局，整合相关部门信息化职能，与新组建的国有控股三门峡崤云信息股份有限公司一同构成全市智慧城市建设的领导主体和实施主体，全面掌控新型智慧城市建设进程；在政策机制上，加强新型智慧城市建设和大数据产业发展相关政策研究，形成一整套制度体系，为新型智慧城市建设和大数据产业发展提供完善的制度保障。

在线管理。充分发挥信息化在城市管理、民生服务、应急管理和政务管理等方面的巨大作用，切实做到“三门峡在线”。实现城市日常管理在线，加快推进智慧城管、智慧交通、智慧水务、智慧社区建设，让城市运行更顺畅，反应更迅速，管理更高效，处置更精准。实现民生服务在线，建好“12345”市民热线平台，实现便民话务服务“一号通”；建设移动终端便民APP，实现便民查询、办理“一点通”；建设财政惠民资金支付系统，实现社保、医保等便捷结算“一卡通”，让群众更好地享受到舒适与便捷。实现应急管理处置在线，加快城市运营指挥中心建设，提升消防、森林防火、防汛抗旱、地质灾害、群体事件等突发事件的智慧化监控和综合处置水平，使突发事件第一时间反应，第一时间处置。实现政务管理监督在线，加快推进移动协同办公系统、互联网+政务建设，让公务员履职尽责时刻在线、步步留痕，把权力关进“数据铁笼”，推动效能革命深入开展，使政务更公开、更阳光、更透明。

（五）持续强化开放带动，不断扩展发展空间。牢固树立开放、竞合、共赢的发展理念，持续扩大对内对外开放，拓展发展新空间。

深入推进央地合作。加强合作意向的跟踪对接，组织专人专班开展服务，建立周报制度，定期集中办公，推动一批合作项目尽快签约落地，加快陕州区航科总部经济基地等项目进度，为全市转型发展提供新的动力和支撑。

持续扩大开放合作。主动融入“一带一路”，持续完善通关体系，加快建设国家级黄金贵金属质检中心，积极与洛阳、西安等丝路沿线城市开展区域合作对接，实现借力发展、错位发展、联动发展。深入推进黄河金三角区域合作，促进产业分工协作，加强生态环境共保共治，推动基本公共服务共建共享。结合产业匹配实际，积极推动与中科院、中南大学等高校和科研院所合作，联合开展技术攻关，推动科技创新实现新突破。

大力发展外经外贸。用好跨境电商综合试验区试点城市机遇，扩大跨境电商产业园规模。积极推进铁路口岸建设，争取年内建成铜精矿进口指定口岸。稳步提升食用菌、鲜果、轮毂等传统出口产品市场份额，组织开展三门峡名优产品丝路行活动，带动更多终端消费品开拓国际市场。鼓励引导优势企业“走出去”，支持中原黄金冶炼厂香港贸易公司发展，进一步扩展外贸合作。

不断优化开放环境。强化“客商第一、服务至上”理念，为项目提供“保姆式”服务，着力营造务实高效的政务环境、公开透明的法治环境、有诺必践的诚信环境、和谐平安的发展环境、尊商富商安商的社会环境。

（六）大力推进改革创新，增强经济发展动能。围绕破解经济社会发展深层次矛盾和问题，深化重要领域和关键环节改革，进一步激发市场活力和社会创造力。

深入推进转型发展攻坚。坚持高端化、绿色化、智能化、融合化，结合实际制定转型攻坚五年行动计划，力争今年在一些重点领域实现实质性突破。

深入推进投融资体制改革。加快市级投融资平台财务整合，推动市投资集团做大做强，力争年内融资100亿元。发挥好各类产业投资基金作用，谋划建立智慧城市、文化旅游、特色农业、黄金珠宝加工、节能环保、城市基础设施等基金，以市场化手段带动社会投资稳步增长。

深入推进国企改革攻坚。进一步完善国有资产管理制度，加快推进政企分开、政事分开，开展国有企业领导选聘工作试点，确保资产保值增值。着力推动功能类、公益类国有企业改革向纵深发展。

深入推进“放管服”改革。优化营商环境，持续降低制度交易成本，继续精简行政审批事项，全面推行权力、责任和效率清单，清理规范中介服务，开展减证便民专项行动。继续深化商事制度改革，实现“双随机、一公开”监管全覆盖。完善“互联网+政务服务”网络，推动行政审批和公共服务事项向网上办事大厅延伸。

深入推进财税金融体制改革。健全县级财政保障机制，继续深化各项预算管理制度改革。抓好税收征管体制改革综合试点工作，建立完善社会保险费征管新机制。依托湖滨农商行和陕州农商行，谋划发展地方金融机构，更好地服务经济发展。

深入推进农村改革。尽快完成“三块地”确权登记发证工作，落实承包土地“三权分置”办法，继续深化水利、林业、供销等改革。

深入推进教育体制改革。落实义务教育县级政府主体责任，进一步整合中心城区教育资源、理顺管理体制，发挥义务教育的基础保障作用。鼓励社会资本投入教育领域，支持民办教育发展。持续推进资源管理、医药卫生、金融、科技、电力、价格、司法等领域改革。

（七）着力改善生态环境，加快推进绿色发展。牢固树立“绿水青山就是金山银山”的理念，加强生态文明建设，努力实现更高质量、更有效率、更加公平、更可持续的发展。

打好环境污染防治攻坚战。继续开展大气治理攻坚，聚焦重点源头、重点行业、重点区域，深入推进科学治污、依法治污、全民治污，全面完成省空气质量改善终期考核目标任务。打响水污染治理攻坚战，严格落实“河长制”，一河一策、水陆统筹，系统推进水污染防治、水生态保护和水资源管理，确保省控断面水质达标。积极推进土壤污染防治，启动土壤污染状况详查，加强土壤污染治理与修复，推进重点地区重金属污染物防治，严格危险废物环境监管。

加快生态环境建设。划定生态保护红线，突出抓好森林、湿地、流域、农田、城市“五大生态”建设，积极争取国家山水林田湖生态保护修复试点城市。继续深入开展小秦岭、黄河湿地生态环境整治，加快实施高铁、高速、国道两侧通道绿化提升工程。完成造林35万亩，森林抚育和改造31万亩。

加大节能减排力度。实施能源消费、水资源消耗总量和强度控制，开展重点行业能效提升工程，实施绿色制造标准化提升行动，推进公共机构建筑节能改造。建立控制污染物排放许可制度，开展低碳试点示范。推进大宗固废综合利用和园区循环化改造，加快传统产业绿色化改造。

（八）始终坚持以人民为中心，切实保障改善民生。集中力量办好省市“十项重点民生工程”等民生实事，让人民群众有更多获得感。

深入推进脱贫攻坚。坚持把脱贫攻坚作为第一民生工程、重大政治任务和头等大事，对照精准扶贫、精准脱贫基本方略，拿出“绣花”功夫，以建立产业扶贫基地（车间）为抓手，以提高贫困户致富技能为根本，综合实施一批产业扶贫、转移就业、易地搬迁、生态补偿、旅游扶贫、电商扶贫、光伏扶贫工程，层层压实责任，激发内生动力，创新方式方法，严格考核奖惩，确保按时完成5万人脱贫、3.9万人易地搬迁脱贫、91个贫困村退出的年度目标，坚决打赢脱贫攻坚战。

统筹解决城建遗留问题。结合去库存，综合运用经济、法律、行政手段，盘活相关房地产项目。采取多种形式，鼓励社会资本参与城市基础设施建设，持续增加城市供暖面积、提高供暖质量，切实解决入学难、如厕难、停车难等与居民生活息息相关的问题，让我们的家园更温暖、更方便、更舒适。

扎实促进就业创业。完善就业创业服务体系，加快创业孵化园和创业基地建设，鼓励大众创业创新，重点支持农民工返乡创业。持续推进全民技能振兴工程，加强劳动技能培训，确保城镇新增就业3.6万人以上。

持续完善社会保障。全面完成机关事业单位养老保险制度改革，加快城乡居民基本医疗保险整合，提高基本养老金标准，提高城乡低保、城乡居民医保财政补助标准，完善医疗、失业、工伤、生育保险市级统筹。加强农村低保与扶贫政策有效衔接，落实特困人员供养制度和残疾人两项补贴，切实兜住困难群体民生底线，使困难群众心里有温暖、生活有奔头。

积极发展社会事业。扎实推进第三期学前教育行动计划和“全面改薄”工作，加快清华园学校、外国语小学改扩建等项目建设。推进健康三门峡建设，深化医疗、医保、医药“三医联动”，深入推进城市公立医院综合改革和县域综合医改，有序开展分级诊疗和家庭医生健康签约服务试点，落实困难群众大病补充保险制度，落实好计划生育家庭奖励优惠政策。加快“五馆一中心”等公共文化服务体系建设，尽快开工建设庙底沟遗址公园，积极推进重大文化工程，发展壮大文化产业，努力打造文化高地。加强公共体育服务体系建设，积极开展全民健身活动，办好市第八届运动会。

不断推进社会治理创新。深入推进企业安全生产标准化建设，确保安全生产形势平稳。坚持源头控制、产管并重、重典治乱，坚决把好人民群众饮食用药安全的每一道关口。完善信访工作机制，健全完善矛盾纠纷排查化解制度，做好重大项目社会稳定风险评估，加强网络舆情管理，确保社会大局和谐稳定。持续推进平安三门峡建设，大力推行治安防控社会化，深化“一村一警”建设，不断提升群众的安全感。深入践行社会主义核心价值观，抓好群众性精神文明创建，培育社会文明新风。

加强国防教育和国防后备力量建设，支持国防和军队改革，支持驻三门峡解放军、武警部队、预备役部队、民兵和人民防空建设，扎实推进军民融合，深入开展双拥共建，让军政军民团结之树根深、枝繁、叶茂！支持工会、共青团、妇联等人民团体更好发挥桥梁纽带作用。继续做好统计、审计、外侨、对台、史志、档案、地震、民族宗教、防灾减灾等工作。

四、加强政府自身建设

复杂多变的经济形势，艰巨繁重的发展任务，对政府工作提出了新的更高要求。我们将按照推进国家治理体系和治理能力现代化的要求，以新精神新理念引领政府改革建设、总揽政府职能转变、贯穿政府各项工作，努力建设人民满意政府。

增强“四个意识”，坚决维护中央权威。始终在思想上政治上行动上同以习近平同志为核心的党中央保持高度一致，切实增强政治意识、大局意识、核心意识、看齐意识。自觉坚持党的基本路线，自觉贯彻党的方针政策，严守政治纪律，恪守政治规矩，保持政治定力，始终做到忠诚、干净、担当，坚决把党中央国务院、省委省政府和市委的决策部署落到实处。

推进依法行政，加快建设法治政府。全面贯彻法治政府建设实施纲要，尊崇法治、敬畏法律、依法办事。坚持公正文明执法，加强执法监督，依法履行职能，把政府活动全面纳入法治轨道。依法接受人大法律监督和工作监督，自觉接受政协民主监督，主动接受社会和舆论监督，严格执行市人大及其常委会的决议、决定，认真听取人大代表、政协委员、民主党派、工商联、无党派人士和各人民团体的意见、建议，所有工作都要体现人民意愿、维护人民利益、接受人民监督。

开展效能革命，大力弘扬“三干”作风。扎实开展 “效能革命”，不断强化政府系统的企业精神和竞争意识，大力倡导干事、干练、干净的务实作风，建立科学高效的工作机制。健全政府工作落实首问负责制、办文办事限时制，进一步压缩会议、精简文件，突出干字当头，真抓实干、埋头苦干，以雷厉风行的实劲、一抓到底的狠劲、锲而不舍的韧劲成就事业、造福人民。强化担当尽责，改进绩效考核办法，完善工作督查制度，健全激励约束机制，鼓励干事，鼓励担当，鼓励奉献，与人民群众同心协力，以实干推动发展，以实干赢得未来！

加强廉政建设，始终保持廉洁本色。认真落实全面从严治党要求，把党风廉政建设和反腐败工作不断引向深入。坚决贯彻落实中央八项规定和省市实施意见，一以贯之纠正“四风”。聚焦重点领域，加强行政监察和审计监督，严肃查处侵害群众利益的不正之风和腐败问题，保持惩治腐败高压态势，持续优化政治生态。

使命重在担当，实干铸就辉煌。让我们更加紧密地团结在以习近平同志为核心的党中央周围，团结带领全市人民，同心同德，拼搏进取，奋力开创三门峡转型发展新局面，以优异成绩迎接党的十九大胜利召开！
